# Mortalidad por desnutrición en el adulto mayor, Colombia, 2014-2016

**DOI:** 10.7705/biomedica.4733

**Published:** 2019-12-30

**Authors:** Magda Ginnette Rodríguez, Elba Giomar Sichacá

**Affiliations:** 1 Grupo de Vigilancia Nutricional, Dirección de Vigilancia y Análisis del Riesgo en Salud Pública, Instituto Nacional de Salud, Bogotá, D.C., Colombia Grupo de Vigilancia Nutricional Dirección de Vigilancia y Análisis del Riesgo en Salud Pública Instituto Nacional de Salud BogotáD.C Colombia

**Keywords:** desnutrición, anciano, mortalidad, certificado de defunción, fenómenos fisiológicos de la nutrición, causas de muerte., Malnutrition, aged, mortality, death certificates, nutritional physiological phenomena, cause of death.

## Abstract

**Introducción.:**

En el contexto de la salud pública y la nutrición, las personas mayores se consideran un colectivo vulnerable. Los programas de atención en salud dan prioridad a los hábitos alimentarios y a la vigilancia del estado nutricional para mejorar su pronóstico vital.

**Objetivo.:**

Estimar los casos de muerte por desnutrición de la población mayor de 65 años en Colombia entre el 2014 y el 2016, para contribuir al análisis y la toma de decisiones en salud encaminadas a mejorar la situación nutricional de esta población.

**Materiales y métodos.:**

Se trata de un estudio descriptivo y retrospectivo en el cual se analizaron los certificados de defunción de los años 2014 a 2016, cuya causa básica de muerte fuesen las deficiencias y anemias nutricionales. Se estimaron las tasas de mortalidad por sexo y departamento de residencia, y las frecuencias de distribución según las variables demográficas.

**Resultados.:**

Las defunciones por desnutrición en Colombia para el adulto mayor en el periodo de estudio, fueron 3.275 (0,5 % del total de muertes). La tasa de mortalidad varió entre 5,4 y 108,3 por cada 100.000 adultos mayores. La mayor mortalidad se presentó en los mayores de 80 años, especialmente en hombres.

**Conclusión.:**

La desnutrición proteico-calórica en los adultos mayores es la causa más frecuente de muerte por desnutrición, seguida de las anemias nutricionales. La mayor mortalidad se presentó en el grupo de edad de mayores de 80 años, y en los departamentos de Amazonas, Guainía y Vaupés, los cuales tienen las mayores tasas para todos los grupos de edad.

## Abstract (en)

**Introduction:**

Older people are positioned within the context of public health and nutrition as a vulnerable group. The priorities of the attention programs focus on eating habits and monitoring their nutritional status to improve their vital prognosis.

**Objective:**

To estimate the cases of death due to malnutrition of the population over 65 years old in Colombia for 2014 to 2016 to contribute to the analysis and decision-making in health to improve the nutritional situation of this population.

**Materials and methods:**

A retrospective descriptive study was carried out analizing death certificates from 2014 to 2016, whose basic cause of death was nutritional deficiencies and anemias. Mortality rates were estimated by sex and department of residence, and distribution frequencies were built based on demographic variables.

**Results:**

There were 3,275 deaths due to malnutrition in Colombia for the elderly in the study period (0.5% of total deaths). The mortality rate varied between 5.4 and 108.3 per 100,000 older adults. The highest mortality occurred in those over 80 years of age, especially in men.

**Conclusion:**

Caloric protein malnutrition in older adults is the most frequent cause of death due to malnutrition, followed by nutritional anemias. The highest mortality occurs in the age group over 80 years of age and the Amazonas, Guainía and Vaupés departments have the highest rates for all age groups.

El envejecimiento es un fenómeno natural y no se debe al aumento de la esperanza de vida. En consecuencia, este proceso se enmarca en posibles limitaciones de las funciones, modificaciones biológicas, funcionales, psicológicas y sociales a lo largo de la vida, desde el nacimiento y hasta la muerte ^1^^,^^2^. 

Los cambios sensoriales, como los del gusto, el olfato y la visión, los problemas dentales, y los problemas gástricos, así como la disminución de la secreción de ácido gástrico, entre otros, pueden modificar o alterar el estado alimentario de los adultos mayores. Esto implica un deterioro del estado nutricional y de salud, por carencias de energía, de macronutrientes y de micronutrientes, como vitaminas y minerales esenciales. Además, se debe tener en cuenta el deterioro de la función cognitiva, el de la capacidad para cuidar de sí mismo y la mayor dependencia, que conllevan cambios psicosociales y ambientales, como aislamiento, soledad y depresión; estos, junto con las carencias nutricionales, podrían incrementar diferentes enfermedades degenerativas y, en algunos casos, llevar a la muerte ^3^^,^^4^.

La desnutrición en la vejez a menudo no se diagnostica. Son pocas las evaluaciones exhaustivas sobre la prevalencia mundial de las diferentes formas de desnutrición y su posible relación directa con la mortalidad en esta etapa de la vida ^5^.

La desnutrición se entiende como el estado patológico debido a una dieta insuficiente de uno o varios nutrientes esenciales o malabsorción de los alimentos ^6^. En el adulto mayor se pueden identificar tres clases de desnutrición:

1. la calórica, que produce pérdida muscular crónica generalizada y ausencia de grasa subcutánea;
2. la proteica, que es un proceso agudo en el que se disminuyen los depósitos de proteína visceral, y
3. la mixta, en la cual coexisten las dos anteriores ^7^^,^^8^

Otros términos que se utilizan para el diagnóstico nutricional en el adulto mayor son la deficiencia nutricional y la anemia. La deficiencia nutricional es el desequilibrio de los nutrientes necesarios para una salud óptima ^9^. La anemia nutricional se caracteriza por la falta de suficientes glóbulos rojos, que son los encargados de transportar una cantidad adecuada de oxígeno a los tejidos ^10^^,^^11^.

Según la Organización Mundial de la Salud (OMS) y la Organización Panamericana de la Salud (OPS), las primeras causas de muerte en los mayores de 60 años en la región de las Américas y en el mundo, en el 2012, fueron las enfermedades isquémicas del corazón (14,51 %), las enfermedades cerebrovasculares (7,67 %), y la demencia y la enfermedad de Alzheimer (6,21 %) ^12^. Aun así, cuando se analizan otras causas de muerte, se encuentran las relacionadas con la nutrición, las cuales debe ser tenidas en cuenta por su relación con otras enfermedades que provocan un deterioro significativo en los adultos mayores hasta llevarlos a la muerte.

Para el 2014, en los Estados Unidos, el 0,21 % de los mayores de 65 años murieron por deficiencias nutricionales, el 0,20 %, por desnutrición, y el 0,25 %, por anemia ^13^. En Colombia, en el año 2008, la tasa de mortalidad por deficiencias nutricionales fue de 34,5 defunciones por 100.000 habitantes ^14^.

Por lo anterior, en los adultos mayores es de vital importancia tener en cuenta la vigilancia del estado nutricional con el fin de mejorar su pronóstico vital y su calidad de vida. Además, esta población va en crecimiento por el aumento de la esperanza de vida, lo que la convierte en un reto para la salud pública y la nutrición comunitaria a nivel nacional y mundial.

Se propuso como objetivo estimar los casos de muerte por desnutrición de la población mayor de 65 años en los departamentos de Colombia del 2014 al 2016, y su situación sociodemográfica, incluyendo estado civil, ocupación, sexo y nivel educativo, para contribuir al análisis y la toma de decisiones en salud encaminadas a mejorar su situación nutricional

## Materiales y métodos

Se trata de un estudio descriptivo y retrospectivo para caracterizar la situación epidemiológica de la mortalidad por desnutrición en los adultos mayores de 65 años en el periodo de 2014 a 2016 en Colombia.

Para la estimación de los casos de muerte por desnutrición, se estudiaron las causas de muerte consignadas en el certificado de defunción; se tomaron los certificados en los que se encontraran la anemia y las deficiencias nutricionales como causa básica, de acuerdo con los códigos de la Clasificación Internacional de Enfermedades (CIE-10) (cuadro 1) ^15^.

**Cuadro 1:** Enfermedades seleccionadas como causas de muerte por desnutrición, con base en la Clasificación internacional de enfermedades, CIE 10

Código	Descripción de la enfermedad
D50	Anemias por deficiencia de hierro
D52	Anemia por deficiencia de folatos
D53	Otras anemias nutricionales
E40	Kwashiorkor
E41	Marasmo nutricional
E42	Kwashiorkor marasmático
E43	Desnutrición proteico-calórica grave no especificada
E63	Otras deficiencias nutricionales

Tomado de: Departamento Administrativo Nacional de Estadísticas, Dirección de Censos y Demografía. Grupo de Estadísticas Vitales

Tabla de consistencia CIE 10-MAE2 2006

Las variables tenidas en cuenta para el análisis fueron: edad del fallecido, sexo, pertenencia étnica, estado civil, departamento de residencia, nivel educativo, área y sitio de defunción, afiliación a la seguridad social en salud y ocupación.

Se calcularon las tasas de muerte por desnutrición según sexo y departamento de residencia, utilizando las proyecciones de población estimadas por el Departamento Administrativo Nacional de Estadísticas (DANE) y publicadas en los censos de población. Para la estimación de la tasa de mortalidad por desnutrición, se consideró la población adulta mayor por grupos quinquenales registrada en el mismo año, y se calcularon las medidas descriptivas para las variables cuantitativas.

### Consideraciones éticas

De acuerdo con la Resolución 08430 de 1993, se realizó un estudio sin riesgo, teniendo en cuenta que se emplearon técnicas y métodos documentales retrospectivos. No se hizo ninguna intervención ni modificación intencionada de las variables biológicas, fisiológicas, sicológicas o sociales.

## Resultados

Las defunciones por desnutrición en Colombia para el adulto mayor en el periodo de estudio, fueron 3.275 (0,5 % del total de muertes). La mayor mortalidad se presentó en el grupo de edad de mayores de 80 años (68,5 %), seguido del grupo de 75 a 79 años (15,5 %). Los hombres mayores de 80 años fueron en quienes se registró la mayor tasa de mortalidad (112 por cada 100.000 adultos mayores) en promedio.

### Grupo de 65 a 69 años

**Figura 1.**
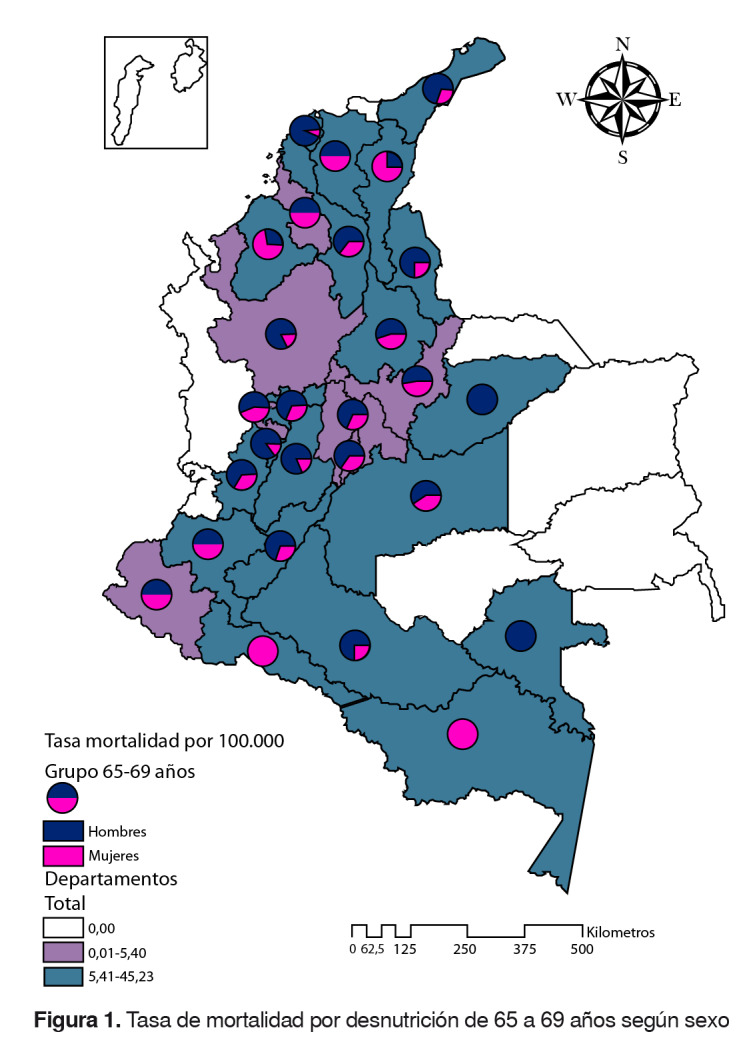
Tasa de mortalidad por desnutrición de 65 a 69 años según sexo

La tasa de mortalidad fue de 5,4 por 100.000 adultos mayores (hombres: 7,3; mujeres: 3,7). Los departamentos con una tasa por encima de la tasa nacional fueron: Vaupés, Amazonas, Casanare, Caquetá, Bolívar, Cesar, Norte de Santander, Huila, Quindío, Tolima, Meta, Atlántico, Magdalena, Valle del Cauca, La Guajira, Caldas, Córdoba, Cauca, Santander y Putumayo. En los departamentos de Casanare y Vaupés se registraron muertes solo en hombres y, en el Amazonas, solo en mujeres. En los departamentos de Arauca, Chocó, Guainía, Guaviare, Vichada y San Andrés y Providencia no se registraron muertes para este grupo de edad (figura 1).

### Grupo de 75 a 74 años

**Figura 2.**
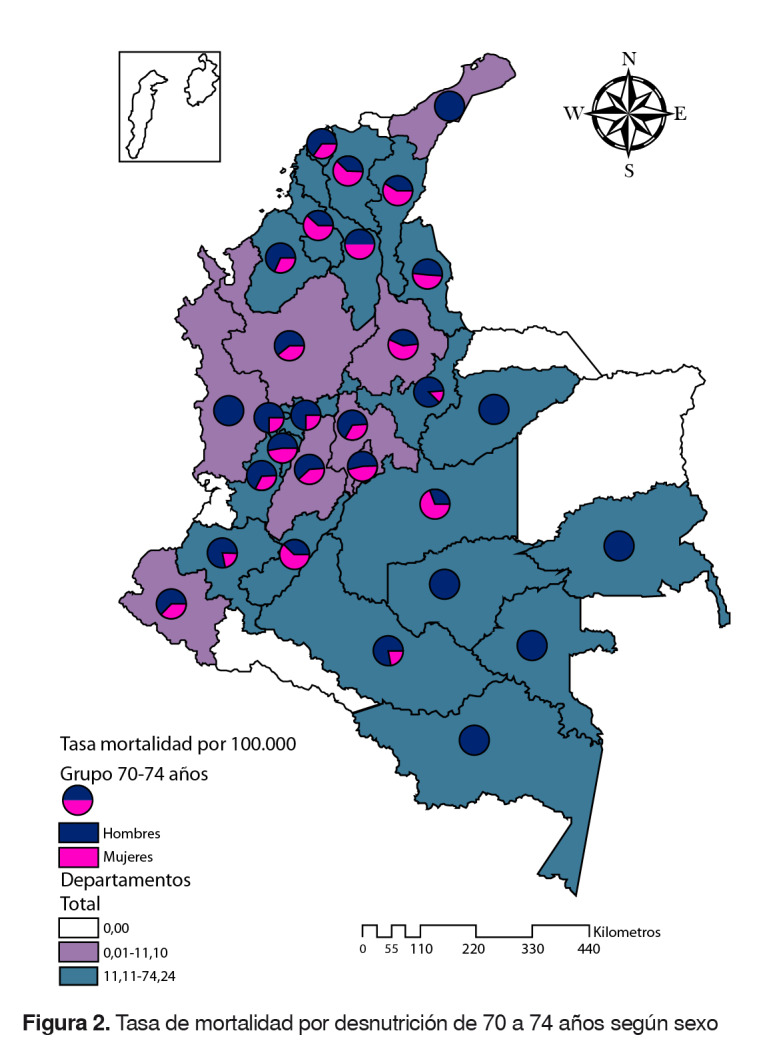
Tasa de mortalidad por desnutrición de 70 a 74 años según sexo

La tasa de mortalidad por desnutrición fue de 11,1 (hombres: 14; mujeres: 8). Los departamentos con una tasa superior a la nacional fueron: Guainía, Vaupés, Amazonas, Sucre, Guaviare, Caquetá, Quindío, Meta, Boyacá, Caldas, Huila, Valle del Cauca, Norte de Santander, Cauca, Cesar, Atlántico, Córdoba, Casanare, Risaralda, Magdalena y Bolívar. En los departamentos de Amazonas, Guainía, Guaviare, Vaupés, La Guajira y Chocó, se presentaron muertes solo en hombres. En los departamentos de Arauca, Putumayo, San Andrés y Providencia, y Vichada no se registraron muertes en este grupo de edad (figura 2).

### Grupo de 75 a 79 años

**Figura 3.**
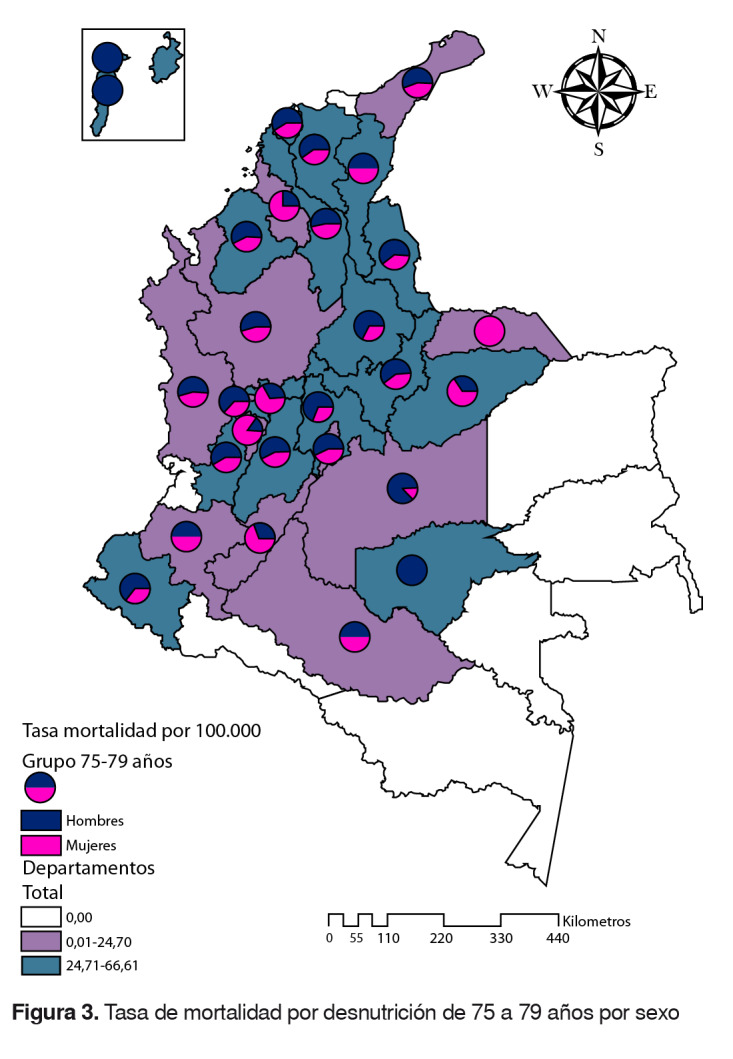
Tasa de mortalidad por desnutrición de 75 a 79 años por sexo

La tasa nacional fue de 24,7 (hombres: 28; mujeres: 22). Los departamentos de Guaviare, Bolívar, Boyacá, Norte de Santander, Cesar, San Andrés y Providencia, Magdalena, Atlántico, Casanare, Valle del Cauca, Córdoba, Nariño, Cundinamarca, Caldas, Tolima y Santander estuvieron por encima de la tasa nacional. En los departamentos de Amazonas, Guainía, Vaupés y Vichada no se registraron muertes en este grupo de edad (figura 3).

### Grupo 80 años y más

**Figura 4.**
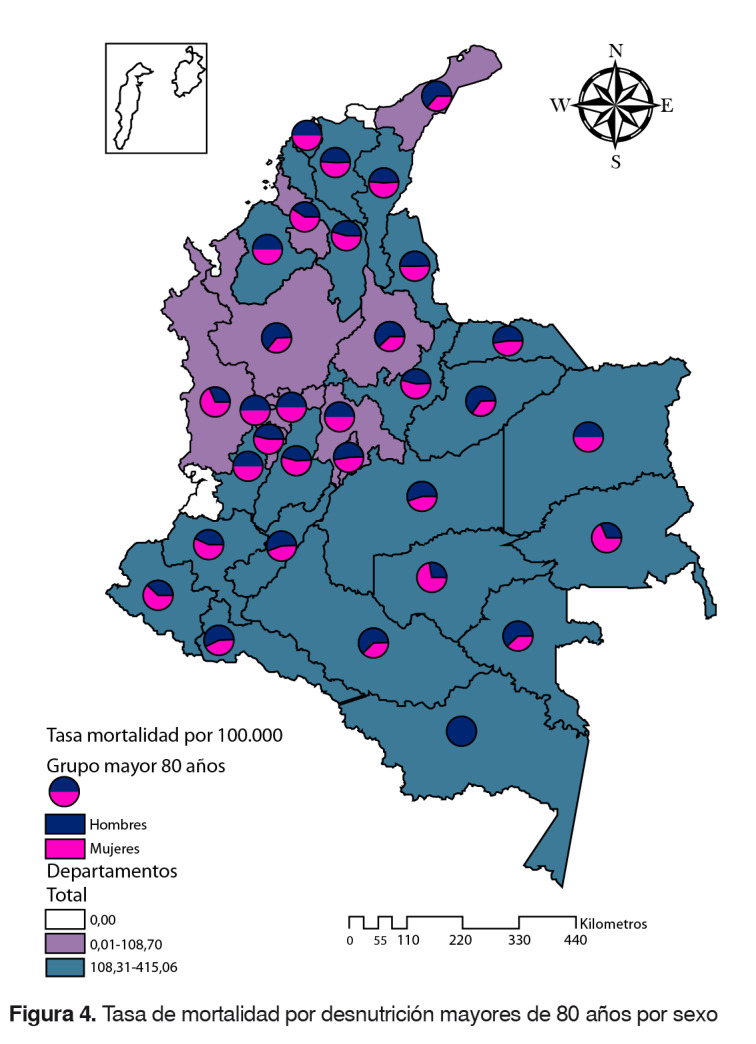
Tasa de mortalidad por desnutrición mayores de 80 años por sexo

La tasa nacional fue de 108,3 por 100.00 adultos mayores de 65 años (hombres: 112; mujeres: 106). Los departamentos con tasas por encima de la nacional fueron: Guainía, Casanare, Amazonas, Norte de Santander, Bolívar, Huila, Vaupés, Cesar, Guaviare, Atlántico, Putumayo, Nariño, Magdalena, Vichada, Cauca, Arauca, Tolima, Valle del Cauca, Córdoba, Boyacá, Caquetá y Meta. En el departamento de San Andrés y Providencia no se registraron muertes por desnutrición en este grupo de edad (figura 4).

Más del 80 % de los adultos mayores estaban casados en los años 2014 (n=1.047) y 2015 (n=993), y la mayor proporción se encontraban viudos en el 2016 (n=460). La mayoría de ellos pertenecía a ‘otros grupos étnicos’ (n=3.024), seguidos de los afrocolombianos con cerca del 5 %. La mayor proporción de los adultos mayores de ambos sexos alcanzó el nivel de primaria (n=1228), y residía principalmente en la cabecera municipal, seguida del área rural dispersa. El 60 % (n=1.965) falleció en instituciones de salud y el 30 % (n=983) en el domicilio. En su mayoría, estaban asegurados con el régimen subsidiado (n=1422) y el 5 % (n=64) no se encontraban asegurados al momento del fallecimiento (cuadro 2).

**Cuadro 2:** Características demográficas por sexo de las defunciones por deficiencias nutricionales de adultos mayores Colombia, 2014-2016

	2014			2015			2016			
Características		(n=1.158)			(n=1.079)					(n=1.038)	
									
Masculino		Femenino		Masculino	Femenino		Masculino	Femenino		
		
	(%)		(%)	(%)		(%)	(%)		(%)	
Grupos de edad (años)										
65-69	4,5		2,5	4,6		2,0	3,3		2,4	
70-74	6,4		4,1	5,6		4,3	4,7		3,4	
75-79	8,3		7,8	8,2		7,6	6,9		7,9	
>80	27,6		38,8	29,8		37,9	30,1		41,3	
Estado civil										
Unión libre >2 años	0,9		1,1	0,5		0,5	4,8		2,2	
Unión libre <2 años	0,0		0,0	0,1		0,0	0,2		0,3	
Separado/divorciado	0,1		0,1	0,0		0,2	1,6		1,0	
Viudo	0,0		0,0	0,0		0,2	10,7		29,0	
Soltero	3,8		2,9	3,1		3,3	8,0		9,5	
Casado	41,6		48,8	44,5		47,5	9,8		4,1	
Sin información	0,4		0,4	0,1		0,2	9,9		8,8	
Pertenencia étnica										
Indígena	0,9		1,2	0,5		0,5	0,9		1,2	
Rom/gitano	0,0		0,1	0,1		0,0	0,0		0,1	
Raizal	0,0		0,0	0,0		0,2	0,0		0,0	
Palenquero	0,0		0,0	0,0		0,2	0,0		0,0	
Afrocolombiano	2,2		3,1	3,1		3,3	2,2		3,1	
Ninguno	42,0		50,5	44,5		47,5	42,0		50,5	
Sin información	0,0		0,0	0,1		0,2	0,0		0,0	
Nivel educativo										
Preescolar	0,6		0,9	1,5		1,0	0,7		0,9	
Primaria	16,0		19,9	18,0		19,6	17,1		22,0	
Secundaria	2,3		2,9	1,3		3,0	2,1		2,2	
Media académica	0,8		1,0	1,1		1,2	1,2		0,7	
Media técnica	0,1		0,1	0,2		0,0	0,1		0,3	
Normalista	0,0		0,3	0,0		0,3	0,1		0,1	
Técnica profesional	0,1		0,2	0,2		0,1	0,1		0,1	
Tecnológica	0,2		0,2	0,1		0,0	0,0		0,0	
Profesional	0,4		0,5	0,4		0,1	0,8		1,0	
Especialización	0,0		0,0	0,1		0,0	0,2		0,0	
Maestría	0,0		0,0	0,1		0,0	0,0		0,0	
Doctorado	0,1		0,0	9,3		12,6	0,0		0,0	
Ninguno	9,7		13,5	0,0		0,0	9,3		12,1	
Sin información	16,5		13,9	16,0		13,9	13,5		15,5	
Área de defunción										
Cabecera municipal	42,9		48,2	43,3		48,6	41,0		49,7	
Centro poblado	0,6		1,6	2,2		1,1	1,1		1,7	
Rural disperso	3,3		3,5	2,7		2,1	2,9		3,5	

Más del 50 % (n=2.352) de los adultos mayores realizaba labores en su hogar, y les seguían las ocupaciones de agricultura (10 %) (n=28) en cultivos transitorios); el 4.3 % (n=141) eran pensionados; otras ocupaciones fueron: vendedor ambulante, cocinero, trabajador doméstico, limpiabotas y obrero de construcción, las cuales correspondieron a menos del 1 %.

En los años 2014 y 2015, como causa de muerte por desnutrición predominó la proteico-calórica no especificada (n=381, n=1.079, respectivamente). Esta fue de 4,3 % (2014, n=45; 2015, n=46) en el grupo de 65 a 69 años, de 5 % y 6,2 % (2014, n=52; 2015, n=67) en el grupo de 70 a 74 años, de 8,6 % (2014, n=89) a 10,3 % (2015, n=111) en el grupo de 75 a 79 años, y de 46,5 % a 44,8 % (2014, n=400; 2015, n=483) en el de los mayores de 80 años. En el año 2016, como causa de muerte por desnutrición predominaron la proteico-calórica grave (n=359) y la anemia nutricional (n=22) para todos los grupos etarios; las enfermedades respiratorias se registraron como causa antecedente a la desnutrición.

## Discusión

La mortalidad por desnutrición para el periodo analizado fue diferente entre hombres y mujeres, en todos los grupos de edad; fue mayor en los hombres y en el grupo de 80 y más años. Al revisar este resultado y compararlo con el estudio de envejecimiento demográfico para Colombia 1951-2020, se podría relacionar con el proceso de transición demográfica, en el cual la mayor longevidad de las mujeres es evidente a lo largo de todo el proceso de envejecimiento de la población, con incrementos sostenidos desde los años 70 hasta la proyección para el 2020. Esto podría significar que hay más mujeres mayores que hombres y que la expectativa de vida de las mujeres es mayor que la de los hombres ^16^.

Este resultado coincide con lo relacionado en el informe de mortalidad evitable en Colombia, en el cual, al comparar el trienio 1998-2000 con el trienio 2009- 2011, se evidenció que, en general, las tasas de mortalidad en hombres fueron más altas que las de mujeres para todos los rangos de edad (58,6 % en hombres y 41,4% en mujeres); estas aumentaron a medida que la edad se incrementaba ^17^.

El que las tasas de mortalidad sean mayores en hombres que en mujeres podría implicar que, por su viudez y el hecho de vivir solas, las mujeres mayores enfrentan problemas específicos relacionados con la edad y el sexo. Cuando estas generaciones de mujeres atravesaron las edades escolares y laborales, no estaba generalizada la educación formal ni tampoco la participación laboral femenina. Por tanto, el nivel educativo de estos adultos mayores es bajo, lo que podría llevar a que la mayoría de las mujeres mayores no cuenten con una actividad laboral productiva, a pesar de haber trabajado durante toda su vida en el hogar, como se relaciona en este análisis ^16^.

Las diferencias en la mortalidad por sexo podrían estar afectadas por el índice de masculinidad que, en el año 2010, descendió progresivamente a lo largo del ciclo de vida, incrementándose constantemente la proporción de mujeres, al pasar de 104 hombres por cada 100 mujeres en el grupo de los menores de 15 años, a 73 hombres por cada 100 mujeres en el grupo de 80 y más años ^2^. Además, en el estudio de mortalidad evitable para Colombia, se encontró en hombres un riesgo de casi el doble de morir por una causa de muerte evitable, al compararlos con las mujeres ^17^.

El 66 % (n=22) de los departamentos está por encima de la tasa de mortalidad nacional para todos los grupos de edad, y los departamentos de Amazonas, Guainía y Vaupés son los que tienen las mayores tasas, prevaleciendo la mortalidad en hombres de la región de la Amazonía. Colombia se encuentra en una etapa intermedia del proceso demográfico, al igual que el resto de Latinoamérica. Esto se podría explicar por el envejecimiento poblacional que no es homogéneo en todo el territorio nacional, lo cual se observa en departamentos como Vaupés, Vichada y Guainía ^2^.

En este sentido, el informe de desigualdades sociales en salud en Colombia refiere que el departamento del Amazonas tiene una tendencia a la disminución de la esperanza de vida en ambos sexos ^18^.

También, se encontró que la tasa de mortalidad aumenta con la edad cuando se superan los 80 años; en esta edad, la desnutrición podría llevar a la pérdida de masa grasa corporal asociada a una cierta pérdida de masa magra, lo cual constituye uno de los problemas nutricionales más importantes en la vejez ^19^, pero no está necesariamente ligada al propio proceso de envejecimiento ^20^, sino a otros factores asociados.

La mitad de los adultos mayores tenía como ocupación las labores del hogar, lo cual se podría relacionar con la pobreza como fenómeno multicausal que afecta de manera distinta a mujeres y hombres. De acuerdo con el análisis de resultados de pobreza monetaria del Departamento Nacional de Planeación, las mujeres presentan condiciones que las hacen más vulnerables al fenómeno de la pobreza, tales como la invisibilidad del trabajo doméstico no remunerado y las restricciones de tiempo que este impone, la discriminación laboral reflejada en menores tasas de participación, las mayores tasas de desempleo y menores salarios, la falta de autonomía económica y la violencia de género, entre otras ^21^^).^

El nivel educativo predominante es la primaria. De acuerdo con el diagnóstico de los adultos mayores en Colombia, se encuentra que, a partir de los 60 años, el promedio es de cinco años estudiados; este es menor en las mujeres y desciende progresivamente hasta ser de alrededor de dos en los más viejos. Respecto a las tasas de analfabetismo, se ha visto que los mayores de 60 años presentan las tasas más altas (23,4 %) ^21^.

La Política Nacional de Envejecimiento y Vejez, 2007-2019, propuesta por el Ministerio de la Protección Social, tiene entre sus retos el envejecimiento demográfico y el envejecimiento femenino, especialmente, además de la erradicación del hambre y de la pobreza extrema en la población de personas mayores ^22^. Estos aspectos deben resaltarse con los resultados obtenidos sobre la viudez femenina, especialmente en el año 2016, el bajo nivel educativo y la dedicación a las labores del hogar, que convierten a los adultos mayores en un grupo social de especial vulnerabilidad.

Se concluye que la mortalidad por desnutrición en todos los grupos de edad analizados es mayor en los hombres que en las mujeres, y que la tasa de mortalidad se incrementa a medida que aumenta la edad.

El departamento de San Andrés y Providencia es el que tiene el menor número de muertes por desnutrición, y las mayores tasas corresponden a los departamentos de Vaupés, Guainía y Vichada.

El comportamiento de la mortalidad por desnutrición se muestra acorde con el envejecimiento demográfico del país, con diferencias entre hombres y mujeres reflejadas, especialmente, en el grupo de 80 y más años.

Se recomienda un análisis a nivel departamental, con el fin de estudiar posibles factores relacionados con la mortalidad por desnutrición del adulto mayor, la inseguridad alimentaria y nutricional, las limitaciones en el acceso a los servicios de salud y el envejecimiento demográfico, de forma que permita brindar información útil para la acción en beneficio de este grupo poblacional.

Como limitación del estudio, se indica que la información analizada proviene directamente de cada hecho vital, teniéndose una cobertura de registro de hechos vitales para el país cercana al 90 %, por lo que puede haber una adecuada validez de dichos resultados. Sin embargo, es importante mencionar que se requiere ampliar este estudio con metodología de ajuste de tasas para reducir un posible subregistro de la información.
